# The Gut Microbiome of Healthy Vietnamese Adults and Children Is a Major Reservoir for Resistance Genes Against Critical Antimicrobials

**DOI:** 10.1093/infdis/jiab398

**Published:** 2021-08-10

**Authors:** Joana Pereira-Dias, Chau Nguyen Ngoc Minh, Chau Tran Thi Hong, To Nguyen Thi Nguyen, Tuyen Ha Thanh, Caroline Zellmer, Hao Chung The, Lindsay Pike, Ellen E Higginson, Stephen Baker

**Affiliations:** 1 University of Cambridge School of Clinical Medicine, Cambridge Biomedical Campus, Cambridge, United Kingdom; 2 Department of Medicine, University of Cambridge School of Clinical Medicine, Cambridge Biomedical Campus, Cambridge, United Kingdom; 3 Oxford University Clinical Research Unit, Ho Chi Minh City, Vietnam; 4 The Wellcome Sanger Institute, Hinxton, Cambridge, United Kingdom

**Keywords:** microbiome, resistome, antimicrobial resistance, Vietnam, pediatric, familial microbiome, urban microbiome

## Abstract

Antimicrobials are a key group of therapeutic agents. Given the animal/human population density and high antimicrobial consumption rate in Southeast Asia, the region is a focal area for monitoring antimicrobial resistance (AMR). Hypothesizing that the gastrointestinal tract of healthy individuals in Vietnam is a major source of AMR genes that may be transferred to pathogens, we performed shotgun metagenomic sequencing on fecal samples from 42 healthy Vietnamese people (21 children and 21 adults). We compared their microbiome profiles by age group and determined the composition of AMR genes. An analysis of the taxonomic profiles in the gut microbiome showed a clear differentiation by age, with young children (age <2 years) exhibiting a unique structure in comparison to adults and older children. We identified a total of 132 unique AMR genes, with macrolide, lincosamide, and streptogramin class resistance genes (*ermB* and *lnuC*) and tetracycline resistance genes being almost ubiquitous across the study population. Notably, samples from younger children were significantly associated with a greater number of AMR genes than other age groups, including key signature genes associated with AMR pathogens (eg, *bla*_CTX-M_, *mph*A). Our data suggest that the gut microbiome of those living in Vietnam, particularly young children, is a substantial reservoir of AMR genes, which can be transferred to circulating enteric pathogens. Our data support the generation of longitudinal cohort studies of those living in urban and rural areas of developing countries to understand the behavior of these AMR reservoirs and their role in generating multidrug-resistant and extensively drug-resistant pathogens.

Antimicrobial resistance (AMR) is an increasing global health crisis, which contributes to morbidity and mortality and places additional burden on healthcare systems [1, 2]. The emergence and spread of AMR bacteria are associated with sustained antimicrobial usage, which occurs in human healthcare and various agricultural sectors [3]. Multiple strategies have been implemented to improve AMR awareness in order to tackle the overuse of antimicrobials [4]. However, the implementation of strict policies regarding antimicrobial usage in low- and middle-income countries (LMICs) remains limited [5].

Vietnam is an LMIC in Southeast Asia with high rates of AMR pathogens. Unregulated antimicrobial consumption has a major impact on AMR [6], and 81.5% of antimicrobials in Vietnam are provided without prescription, an issue arising in both in urban and rural areas [7]. Additionally, up to a third of patients in Vietnamese hospitals are inappropriately prescribed cephalosporins, penicillin, aminoglycosides, and imidazoles [6]. The use/overuse of antimicrobials in Vietnam has been suggested to maintain the circulation of specific AMR genes in the gut microbiome of healthy members of the Vietnamese population [8, 9]. Of particular concern is the usage of specific classes of broad-spectrum antimicrobials such as third-generation cephalosporins, fluoroquinolones, and macrolides. Over the past 2 decades Vietnam has observed increasing AMR in sentinel gram-negative bacteria associated with both hospital- and community-acquired infections [10]. One of the best examples has been the sequential emergence of resistance to fluoroquinolones and third-generation cephalosporins in *Shigella sonnei* [11]. This trend has been facilitated by self-transmissible plasmids carrying genes encoding extended-spectrum β-lactamases (ESBLs), which can then acquire additional genes encoding resistance to macrolides and aminoglycosides, leading to the emergence of extensively drug-resistant (XDR) *Shigella sonnei* [12].

Our previous work suggested that antimicrobial exposure, specifically the fluoroquinolones, may facilitate the transfer of AMR plasmids from commensal Enterobacteriaceae to infecting *Shigella* during treatment [12]. Therefore, we suggested that gut microbiota in the Vietnamese population may constitute a vast reservoir of AMR genes, which may be transferred to infecting pathogens via their associated vehicles and compound the mounting AMR crisis. This observation is particularly worrisome given the potential rollout of mass drug administration in LMICs using various antimicrobial classes (including ciprofloxacin and azithromycin) that are likely to favor the sustainment of multiple AMR genes and organisms and cause disruption to the microbiome [13].

Given this potential scenario, the paucity of microbiome and AMR gene burden data from LMICs is stark. Such data could contribute to publicly available resources for clinical and scientific stakeholders. Similar data can be useful in numerous instances, such as microbiome-targeted and microbiome-derived therapeutics for a range of communicable and noncommunicable diseases. To date, there are no studies exploiting shotgun metagenomics to generate a comprehensive understanding of the human gut biodiversity and assess the composition of the AMR gene reservoir in healthy Vietnamese people. Following our previous work and hypothesizing that the gut microbiome is a major reservoir of AMR genes in this location, we recruited a cohort of Vietnamese adults and children to generate a detailed overview of the gut microbiome and antimicrobial resistome in these individuals.

## MATERIALS AND METHODS

### Study Population

Forty-two stool samples were collected from 21 adults and 21 children in Ho Chi Minh City, Vietnam, at a single time point. Eligible participants were healthy adults aged ≥18 years working at the Oxford University Clinical Research Unit in Ho Chi Minh City (OUCRU), and children aged 9–60 months with a legal guardian working at OUCRU. The age range was broad given the accessibility to the study population and the same age group of other ongoing studies. Nine months was selected as we recruited children who had been weaned on solid food for at least 3 months. Eligibility was restricted to participants without any known parasitic, bacterial, or viral gastrointestinal infection (screened by Luminex xTag assay), with no record of diarrheal episodes for the last 6 months prior to enrollment. Participants could not have taken antimicrobials in the 3 months prior to recruitment. Additional exclusion criteria included history of a chronic intestinal disease (eg, Crohn disease, ulcerative colitis, celiac disease, irritable bowel syndrome, stomach ulcers, and colorectal cancer) and chronic autoimmune disease or allergies. Informed consent was obtained from all participants or their legal parent/guardian. Recruitment was coordinated by a sample manager who ensured participant and specimen anonymity to other study staff. Ethical approval for this study was obtained from the Oxford Tropical Research Ethics Committee (OxTREC, ID: 505–17). All recruited children (median age, 23 months [interquartile range, 9–37 months]) had been weaned onto a solid food diet for at least 3 months prior to recruitment. All recruited adults (median age, 35 years) were reported to have an omnivorous diet.

### Stool DNA Extraction and Sequencing

Stool was collected noninvasively using Protocult Collection Device (Ability Building Community, Rochester, Minnesota) and frozen at –80°C for up to 14 days. Specimens were homogenized in phosphate-buffered saline at 100 mg/mL and DNA was extracted using the FastDNA Spin Kit for Soil (MP Biomedicals, Solon, Ohio), in accordance with the manufacturer’s instructions. DNA samples were sequenced at Wellcome Trust Sanger Institute (Cambridge, United Kingdom), generating paired end reads of 125 bp per sample. Sequence data are publicly available in the European Nucleotide Archive with accession numbers ERS1865478–ERS1865519.

### Microbiome Diversity

Raw sequence reads were screened for human reads, which were then filtered out by using Bowtie2 version 2.2.3 [14]. The remaining reads were then classified against the uhgg_kraken2-db prokaryotic genome database using Kraken2 version 2.08-beta [15, 16]. To estimate abundance, Kraken2 output files were used to run Bracken version 2.5 at the species and phylum level [17]. After samples were rarefied based on the sample with the lowest total reads, biodiversity analysis was carried out at species level using R version 4.0.2 with vegan version 2.5–6 package [18]. Statistical analysis of α-diversity was carried out using pairwise Wilcoxon rank-sum test.

### AMR Analysis

AMR genes were detected from raw sequence files using SRST2 version 2 and the ARGANNOT database [19], before being quantified and normalized based on gene length and size using reads per kilobase per million mapped reads (RPKM) [20]. For comparative analysis, the median and range of the total amount of ARGs in each age group were calculated and compared by using the Wilcoxon rank-sum test. Comparisons between age groups for each individual antimicrobial class were measured using a 2-tailed Fisher exact test.

## RESULTS

### Human Gut Biodiversity

**Figure 1.**
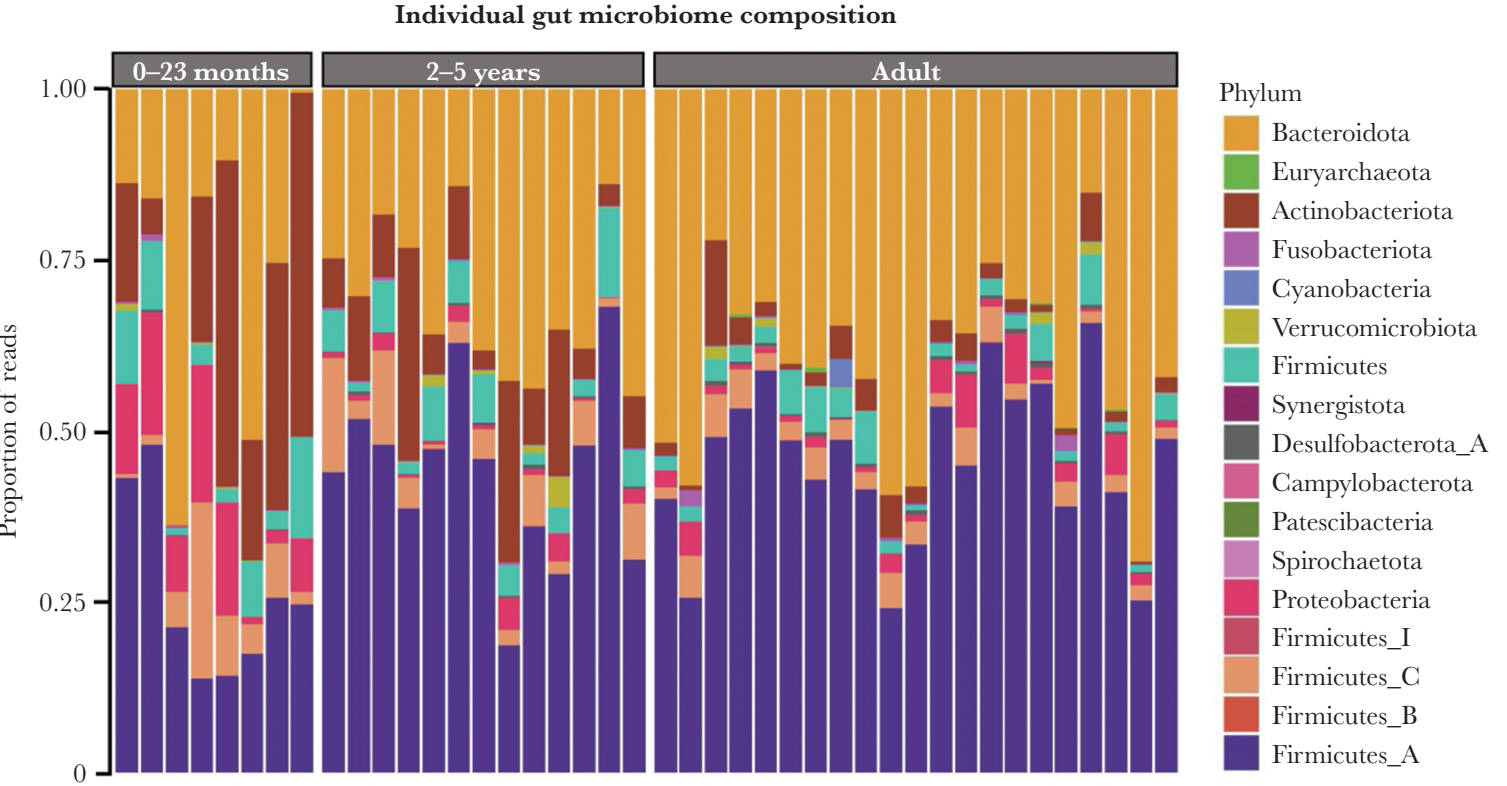
Gut microbiome composition in fecal samples from healthy Vietnamese participants. Bar chart by individual outlining the composition of the fecal microbiome by proportion of sequencing reads (%) at phylum level by age group: 0–23 months, 2–5 years, and adults. Each phylum has a unique color code (see key).

Fecal samples were prospectively collected from 21 healthy adult and 21 healthy child (<5 years) donors in Ho Chi Minh City, Vietnam. Total DNA was extracted from the fecal samples and subjected to Illumina sequencing, generating an average of 12.4 million reads per sample. Due to the known changes in microbiome composition that occur in children after infancy, participants were separated into 3 age groups for analysis: 0–23 months (n = 8), 2–5 years (n = 13), and adults (n = 21). The relative abundance of the different phyla between age groups after filtering is shown in Figure 1. Overall, the relative abundance of Bacteroidetes was greater in the adult samples than in the child samples. Additionally, adults and older children also had a higher abundance of Firmicutes A; in contrast, younger children had a high abundance of Proteobacteria and Actinobacteria.

**Figure 2.**
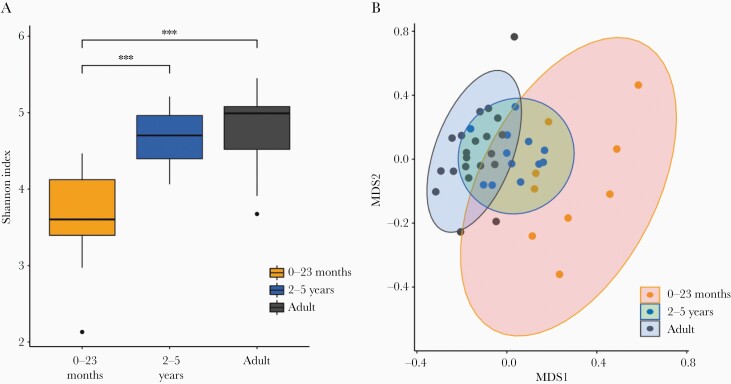
Microbial diversity in fecal samples from healthy Vietnamese participants. Microbial species diversity stratified in 3 different age groups: 0–23 months, 2–5 years, and adults. *A*, Box plots showing sample evenness measured using Shannon index. *B*, Between-sample dissimilarity measured by using Bray–Curtis dissimilarity. ***P < .05 (pairwise Wilcoxon test). Abbreviation: MDS, multidimensional scaling.

To further investigate differences in the microbial composition, data were compared at the species level. First, a within-sample composition (α-diversity, Shannon index) analysis was performed to compare the overall number of the different taxonomic groups in each sample (richness) and how evenly they were distributed within the sample (evenness). A pairwise Wilcoxon rank-sum test revealed a significant difference in α-diversity between the 2 pediatric groups (0–23 months and 2–5 years) (*P < *.0001) and between those aged 0–23 months and adults (*P < *.0001). Conversely, no significant difference was observed between the age group 2–5 years and adults (Figure 2A). The existent dissimilarity in α-diversity of the gut microbiome of those aged 0–23 months, when compared to the α-diversity of the other 2 groups (2–5 years and adults), demonstrated that the composition and abundance of the taxonomic groups in those aged 0–23 months differed between the other 2 groups.

Further analysis comparing species dissimilarity between microbial communities (β-diversity) was conducted using the Bray–Curtis index (Figure 2B). While diversity in the gut microbiome samples from the age group 0–23 months was widely dispersed, the remaining 2 groups were less well dispersed and exhibited significant overlap, further suggesting little difference in bacterial composition between these 2 groups. Moreover, the microbial diversity in samples from individuals in the age group 2–5 years had similarities with both the other age groups.

### Antimicrobial Resistance Gene Carriage

**Figure 3.**
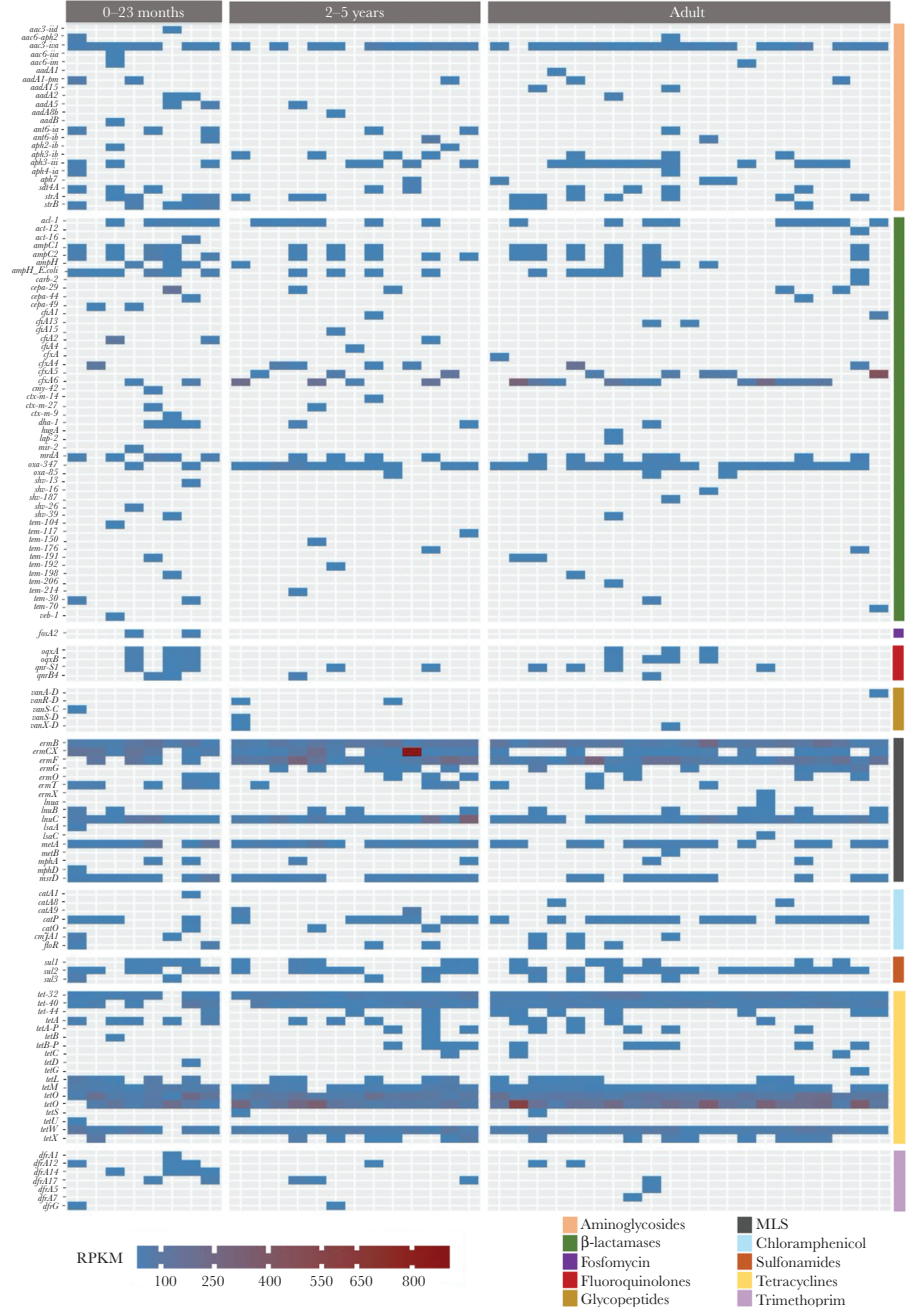
Relative abundance of antimicrobial resistance (AMR) genes in fecal samples from healthy Vietnamese participants. Plot generated by reads per kilobase per million mapped reads (RPKM). Samples are stratified by age group (0–23 months, 2–5 years, and adults), and each AMR gene (y-axis) is organize by antimicrobial class (aminoglycosides, β-lactamases, fosfomycin, fluoroquinolones, glycopeptides, MLS [macrolide, lincosamide, and streptogramin], chloramphenicol, sulfonamides, tetracyclines, and trimethoprim).

Raw sequence reads were screened for the presence of genes conferring AMR, and the relative abundance for each individual AMR gene was calculated by RPKM. A heatmap was used to represent the relative abundance of each AMR gene (Figure 3). In total, 132 AMR genes were identified within the shotgun metagenomic sequencing data. Macrolide, lincosamide, and streptogramin (MLS) resistance genes, including *ermB* and *lnuC*, and the tetracycline resistance genes *tet*-*32*, *tetM*, *tetO*, *tetQ*, and *tetW* were identified in >95% of the participant samples. However, among all detected AMR genes, the β-lactamase encoding genes exhibited the greatest diversity; 48 unique β-lactamase genes were identified. This translated into all participants possessing a minimum of 2 unique β-lactamase encoding genes.

From the 132 AMR genes detected, half (66/132) were only found in 1 or 2 participants. These included a large proportion of the 48 β-lactamase encoding genes; more than half (32/48 [66.67%]) of these were only present in <5% (2 participants) of the study population. For resistance to other antimicrobial classes, we found that 42.86% (3/7), 54.54% (12/22), 57.14% (4/7), 64.70% (11/17), and 72.22% (x/x) 13/18 of trimethoprim, aminoglycoside, chloramphenicol, MLS, and tetracycline resistance genes, respectively, were present in >2 participants. Last, fluoroquinolone resistance genes were the only class for which all 4 of the detected genes (*oqxA*, *oqxB*, *qnr-S1*, and *qnrB4*) were present in at least 10% of the population. Several highly relevant genes associated with XDR organisms were identified in healthy individuals; these included *bla*_CTX-M_ β-lactamases, *mph*A/D azithromycin resistance genes, and the *aac* gentamicin resistance genes. Notably, the *bla*_CTX-M_ genes were only found in children, whereas the azithromycin and gentamicin resistance genes were found in both adults and children.

An overall comparison of AMR gene abundance revealed that infants belonging to the age group 0–23 months possessed a greater number of AMR genes compared to the other 2 age groups. The median number of detected AMR genes was 31 (range, 21–41) for the age group 0–23 months and 25 (range, 16–35) for the age group 2–5 years. Adults possessed a median of 21 (range, 16–39) AMR genes per participant. A Wilcoxon rank-sum test suggested that the samples from the age group 0–23 months possessed significantly more AMR genes than the other age groups (*P < *.05). Investigating this trend in more detail, we found that fosfomycin resistance genes were only present in children the younger age group. Additionally, children in the age group 0–23 months more commonly possessed 3 or more fluoroquinolone resistance genes.

## Discussion

Collectively, AMR surveillance and microbiome characterization are practical approaches for establishing a baseline measure of AMR genes circulating within a given population in LMICs, which can then be used to inform strategies to reduce AMR [21]. We have previously found that gram-negative enteric pathogens are common in Vietnam and appear to have sustained access to a variable reservoir of AMR genes. Therefore, hypothesizing that the gastrointestinal microbiota in the human Vietnamese population was likely a major reservoir of AMR genes that can be transferred to pathogens, we conducted a pilot study which sought to assess the composition of the microbiome and AMR gene in Vietnamese participants of different age groups.

We found that there was a significant difference in the composition of gut microbiome between those aged 0–23 months and the other 2 older age groups. This result corroborates previous findings that identified a lower microbiome diversity in children aged <12 months when compared to older children and adults [22, 23]. Additionally, the gut microbiome profile in children >2 years was more closely related to the adults than children aged <2 years. Previous studies have reported the impact of breastfeeding on the gut microbiome in the first years of life and changes that occur after weaning [24]. We suggest that the weaning process induces a rapid maturation into a comparatively stable and adult-like microbiome. Future investigations with larger cohorts of children aged <24 months with more granular data regarding diet and birth route are required to identify microbiome trends associated with delivery and dietary changes in LMICs.

We additionally observed that AMR genes were present in every sample and that the *ermB*, which is transposon-associated and confers resistance to macrolides, was identified in the entire study population [25]. The high detection of this gene in Vietnam is consistent with previous studies that reported the *ermB* gene to be the most abundant *erm* gene in China, Denmark, and Spain [26]. The presence of tetracycline resistance genes such as *tetM*, *tetO*, *tetQ*, and *tetW *in >95% of the study population may be associated with the large amount of tetracyclines used in agriculture [27–30].

We detected the presence of fluoroquinolone resistance genes in >10% of the samples; this rate of detection may be associated with the horizontal transfer of plasmid-mediated quinolone resistance genes via co-selection from the use of other nonfluoroquinolone antimicrobials [31]. This observation is of particular concern as fluoroquinolones are broad-spectrum antimicrobials that are widely used for a range of infectious diseases [32]. Alternative methods, such as Hi-C technology, that can link AMR genes with specific organisms may assist in understanding how these genes spread via co-selection [33]. Additionally, cephalosporins are a commonly used antimicrobial class in Vietnam [6] and we detected *bla*_CMY_, *bla*_SHV,_ and *bla*_TEM_ in the fecal samples. More specifically, we identified *bla*_CTX-M_, a group of ESBL genes that have been commonly found in a range of enteric pathogens, including *Shigella* and *Salmonella* [12, 34], in several samples from young children. The diversity of AMR genes identified corroborates with a previous study conducted in untreated sewage from countries with different economic backgrounds, which concluded that the global distribution of AMR genes is not only dependent on the socioeconomic status of each country, but also that Vietnam is one of the countries with the most divergent distribution of AMR genes [35]. Indeed, our key observation was that children aged <2 years had the greatest amount of AMR genes in comparison to others. The reason for this is unknown, but we suggest it is because this group is the most likely to receive antimicrobials when ill and the most likely to be infected with an enteric pathogen [36, 37].

This study had several limitations, including the small number of samples and minimal metadata collected. Previous studies have revealed the impact that the environment, hygiene practices and antimicrobial administration have on the share of resistance genes in a population, particularly within children [38–40]. Although we did not collect this information in our pilot study, future research investigating a larger cohort with more comprehensive metadata collection will be needed to understand the impact of the external pressures such as the lived environment and antimicrobial usage on the reservoir of AMR genes in the gut.

Our work contributes to the overall understanding of the gut microbiome and resistome of adults and children in a Southeast Asian LMIC. We observed that while tetracycline and erythromycin resistance genes were found to be highly prevalent across all age groups in this population, a significant difference in microbial diversity and the abundance of AMR genes was noted between the age groups. Our data highlight the high abundance of AMR genes in the gut microbiome of healthy adults and children, which may play a role in driving antibiotic resistance in enteric pathogens. We need to better understand the mechanisms driving microbial diversity and the selective pressure maintaining resident AMR genes in people from LMICs. We suggest large longitudinal cohort studies of populations living in urban and rural areas of LMICs to understand the behavior of these AMR reservoirs and their role in creating multidrug-resistant and XDR pathogens.

## Notes

***Acknowledgments.*** The authors wish to thank all participants and their parents/guardians for their participation in the study. We are further grateful to the Wellcome Sanger Institute Sequencing Pipelines team for sequencing assistance and to the Wellcome Sanger Institute Pathogen Informatics team for bioinformatics support.

***Disclaimer.*** The funders had no role in the design and conduct of the study; collection, management, analysis, and interpretation of the data; preparation, review, or approval of the manuscript; and decision to submit the manuscript for publication.

***Financial support.*** This work was funded by a Wellcome International Training Fellowship (award number 218726/Z/19/Z to H. C. T.) and a Wellcome Senior Research Fellowship (award number 215515/Z/19/Z to S. B.).

***Potential conflicts of interest.*** All authors: No reported conflicts of interest.

Presented in part: 15th Asian Conference on Diarrhoeal Disease and Nutrition, Dhaka, Bangladesh, January 2020.

## References

[1] van Duin D, Paterson DL. Multidrug-resistant bacteria in the community: trends and lessons learned. Infect Dis Clin North Am 2016; 30:377–390. 27208764 10.1016/j.idc.2016.02.004 PMC5314345

[2] Medernach RL, Logan LK. The growing threat of antibiotic resistance in children. Infect Dis Clin North Am 2018; 32:1–17. 29406971 10.1016/j.idc.2017.11.001 PMC5927609

[3] The antibiotic alarm. Nature 2013; 495:141. 10.1038/495141a 23495392

[4] Nguyen KV, Thi Do NT, Chandna A, et al. Antibiotic use and resistance in emerging economies: a situation analysis for Viet Nam. BMC Public Health 2013; 13:1158. 24325208 10.1186/1471-2458-13-1158 PMC4116647

[5] Kakkar M, Chatterjee P, Chauhan AS, et al. Antimicrobial resistance in South East Asia: time to ask the right questions. Glob Health Action 2018; 11:1483637. 29921172 10.1080/16549716.2018.1483637 PMC6008583

[6] Thu TA, Rahman M, Coffin S, Harun-Or-Rashid M, Sakamoto J, Hung NV. Antibiotic use in Vietnamese hospitals: a multicenter point-prevalence study. Am J Infect Control 2012; 40:840–4. 22341530 10.1016/j.ajic.2011.10.020

[7] Nga DTT, Chuc NTK, Hoa NP, et al. Antibiotic sales in rural and urban pharmacies in northern Vietnam: an observational study. BMC Pharmacol Toxicol 2014; 15:1–10. 24555709 10.1186/2050-6511-15-6 PMC3946644

[8] Dat VQ, Toan PK, Van Doorn HR, Thwaites CL, Nadjm B. Purchase and use of antimicrobials in the hospital sector of Vietnam, a lower middle-income country with an emerging pharmaceuticals market. PLoS One 2020; 15:1–17. 10.1371/journal.pone.0240830 PMC7575121 33079967

[9] Vu Thi Ngoc B, Thanh L, Thai P, et al. An exploration of the gut and environmental resistome in a community in northern Vietnam in relation to antibiotic use. Antimicrob Resist Infect Control 2019; 8:194. 31798840 10.1186/s13756-019-0645-9 PMC6883630

[10] Holt KE, Thieu Nga TV, Thanh DP, et al. Tracking the establishment of local endemic populations of an emergent enteric pathogen. Proc Natl Acad Sci U S A 2013; 110:17522–7. 24082120 10.1073/pnas.1308632110 PMC3808646

[11] Chung The H, Boinett C, Pham Thanh D, et al. Dissecting the molecular evolution of fluoroquinolone-resistant *Shigella sonnei*. Nat Commun 2019; 10:4828. 31645551 10.1038/s41467-019-12823-0 PMC6811581

[12] Thanh Duy P, Thi Nguyen TN, Vu Thuy D, et al. Commensal *Escherichia coli* are a reservoir for the transfer of XDR plasmids into epidemic fluoroquinolone-resistant *Shigella sonnei*. Nat Microbiol 2020; 5:256–64. 31959970 10.1038/s41564-019-0645-9 PMC6992430

[13] Mack I, Sharland M, Berkley JA, Klein N, Malhotra-Kumar S, Bielicki J. Antimicrobial resistance following azithromycin mass drug administration: potential surveillance strategies to assess public health impact. Clin Infect Dis 2020; 70:1501–8. 31633161 10.1093/cid/ciz893 PMC7670997

[14] Langmead B, Salzberg SL. Fast gapped-read alignment with Bowtie 2. Nat Methods 2012; 9:357–9. 22388286 10.1038/nmeth.1923 PMC3322381

[15] Wood DE, Lu J, Langmead B. Improved metagenomic analysis with Kraken 2. Genome Biol 2019; 20:257. 31779668 10.1186/s13059-019-1891-0 PMC6883579

[16] Almeida A, Nayfach S, Boland M, et al. A unified catalog of 204,938 reference genomes from the human gut microbiome. Nat Biotechnol 2021; 39:105–14. 32690973 10.1038/s41587-020-0603-3 PMC7801254

[17] Lu J, Breitwieser FP, Thielen P, Salzberg SL. Bracken: estimating species abundance in metagenomics data. PeerJ Comput Sci 2017; 3:e104. 10.7717/peerj-cs.104 PMC12016282 40271438

[18] Oksanen J. Vegan: ecological diversity. R package version 24-4. 2017. https://cran.r-project.org/package=vegan. Accessed 16 December 2021.

[19] Gupta SK, Padmanabhan BR, Diene SM, et al. ARG-ANNOT, a new bioinformatic tool to discover antibiotic resistance genes in bacterial genomes. Antimicrob Agents Chemother 2014; 58:212–20. 24145532 10.1128/AAC.01310-13 PMC3910750

[20] Guitor AK, Raphenya AR, Klunk J, et al. Capturing the resistome: a targeted capture method to reveal antibiotic resistance determinants in metagenomes. Antimicrob Agents Chemother 2020; 64:1–18. 10.1128/AAC.01324-19 PMC7187591 31611361

[21] Allcock S, Young EH, Holmes M, et al. Antimicrobial resistance in human populations: challenges and opportunities. Glob Heal Epidemiol Genomics 2017; 2:e4. 10.1017/gheg.2017.4 PMC5732576 29276617

[22] Stewart CJ, Ajami NJ, O’Brien JL, et al. Temporal development of the gut microbiome in early childhood from the TEDDY study. Nature 2018; 562:583–8. 30356187 10.1038/s41586-018-0617-x PMC6415775

[23] Laursen MF, Bahl MI, Michaelsen KF, Licht TR. First foods and gut microbes. Front Microbiol 2017; 8:1–8. 28321211 10.3389/fmicb.2017.00356 PMC5337510

[24] Ho NT, Li F, Lee-Sarwar KA, et al. Meta-analysis of effects of exclusive breastfeeding on infant gut microbiota across populations. Nat Commun 2018; 9:4169. 30301893 10.1038/s41467-018-06473-x PMC6177445

[25] Leclercq R . Mechanisms of resistance to macrolides and lincosamides: nature of the resistance elements and their clinical implications. 2002; 34:482–92. 10.1086/324626 11797175

[26] Hu Y, Yang X, Qin J, et al. Metagenome-wide analysis of antibiotic resistance genes in a large cohort of human gut microbiota. Nat Commun 2013; 4:2151. 23877117 10.1038/ncomms3151

[27] Lekagul A, Tangcharoensathien V, Yeung S. Patterns of antibiotic use in global pig production: a systematic review. Vet Anim Sci Elsevier 2019; 7:100058. 10.1016/j.vas.2019.100058 PMC7386699 32734079

[28] Kodimalar K, Rajini RA, Ezhilvalavan S, Sarathchandra G. A survey of chlortetracycline concentration in feed and its residue in chicken egg in commercial layer farms. J Biosci 2014; 39:425–31. 24845506 10.1007/s12038-014-9425-0

[29] Pham DK, Chu J, Do NT, et al. Monitoring antibiotic use and residue in freshwater aquaculture for domestic use in Vietnam. Ecohealth 2015; 12:480–9. 25561382 10.1007/s10393-014-1006-z PMC4623066

[30] Taylor P, Reeder R. Antibiotic use on crops in low and middle-income countries based on recommendations made by agricultural advisors. CABI Agric Biosci 2020. doi:10.1186/s43170-020-00001-y.

[31] Thi L, Vien M, Ngoc N, et al. The co-selection of fluoroquinolone resistance genes in the gut flora of Vietnamese children. PLoS One 2012; 7:e42919. 22937000 10.1371/journal.pone.0042919 PMC3427306

[32] Redgrave LS, Sutton SB, Webber MA, Piddock LJV. Fluoroquinolone resistance: mechanisms, impact on bacteria, and role in evolutionary success. Trends Microbiol 2014; 22:438–45. 24842194 10.1016/j.tim.2014.04.007

[33] Oniciuc EA, Likotrafiti E, Alvarez-Molina A, Prieto M, Santos JA, Alvarez-Ordóñez A. The present and future of whole genome sequencing (WGS) and whole metagenome sequencing (WMS) for surveillance of antimicrobial resistant microorganisms and antimicrobial resistance genes across the food chain. Genes 2018; 9:1–28. 10.3390/genes9050268 PMC5977208 29789467

[34] Duong VT, The HC, Nhu TDH, et al. Genomic serotyping, clinical manifestations, and antimicrobial resistance of nontyphoidal *Salmonella* gastroenteritis in hospitalized children in Ho Chi Minh City, Vietnam. J Clin Microbiol 2020; 58:e01465-20. 32907994 10.1128/JCM.01465-20 PMC7685882

[35] Hendriksen RS, Munk P, Njage P, et al; Global Sewage Surveillance Project Consortium. Global monitoring of antimicrobial resistance based on metagenomics analyses of urban sewage. Nat Commun 2019; 10:1124. 30850636 10.1038/s41467-019-08853-3 PMC6408512

[36] Nhi LTQ, De Alwis R, Lam PK, et al. Quantifying antimicrobial access and usage for paediatric diarrhoeal disease in an urban community setting in Asia. J Antimicrob Chemother 2018; 73:2546–54. 29982636 10.1093/jac/dky231 PMC6105870

[37] Duong VT, Tuyen HT, Van Minh P, et al. No clinical benefit of empirical antimicrobial therapy for pediatric diarrhea in a high-usage, high-resistance setting. Clin Infect Dis 2018; 66:504–11. 29029149 10.1093/cid/cix844 PMC5850041

[38] Collignon P, Beggs JJ, Walsh TR, Gandra S, Laxminarayan R. Anthropological and socioeconomic factors contributing to global antimicrobial resistance: a univariate and multivariable analysis. Lancet Planet Health 2018; 2:e398–405. 30177008 10.1016/S2542-5196(18)30186-4

[39] Ramay BM, Caudell MA, Cordón-Rosales C, et al. Antibiotic use and hygiene interact to influence the distribution of antimicrobial-resistant bacteria in low-income communities in Guatemala. Sci Rep 2020; 10:13767. 32792543 10.1038/s41598-020-70741-4 PMC7426860

[40] Doan T, Worden L, Hinterwirth A, et al. Macrolide and nonmacrolide resistance with mass azithromycin distribution. N Engl J Med 2020; 383:1941–50. 33176084 10.1056/NEJMoa2002606 PMC7492079

